# Successful microbial colonization of space in a more dispersed manner

**DOI:** 10.1038/s43705-021-00063-7

**Published:** 2021-11-20

**Authors:** Xiaonan Liu, Miaoxiao Wang, Yong Nie, Xiao-Lei Wu

**Affiliations:** 1 College of Engineering, Peking University, 100871 Beijing, China; 2 Institute of Ocean Research, Peking University, 100871 Beijing, China; 3 Institute of Ecology, Peking University, 100871 Beijing, China

**Keywords:** Microbial ecology, Microbial ecology

## Abstract

Many organisms live in habitats with limited nutrients or space, competition for these resources is ubiquitous. Although spatial factors related to the population’s manner of colonizing space influences its success in spatial competition, what these factors are and to what extent they influence the outcome remains underexplored. Here, we applied a simulated competitive model to explore the spatial factors affecting outcomes of competition for space. By quantifying spatial factors, we show that colonizing space in a more dispersed manner contributes to microbial competitive success. We also find that the competitive edge deriving from a more dispersed manner in colonization can compensate for the disadvantage arising from either a lower growth rate or lower initial abundance. These findings shed light on the role of space colonization manners on maintaining biodiversity within ecosystems and provide novel insights critical for understanding how competition for space drives evolutionary innovation.

## Introduction

Competition is a ubiquitous phenomenon observed for both microorganisms and macro-organisms [1, 2]. It is considered to represent a key factor driving biodiversity [3, 4] and evolution [5, 6]. Competition often occurs when individual organisms compete for identical resources [7–10]. It is characterized by the consumption of a limiting resource by one population, resulting in a decrease in the fitness of its competitors. Nutrient and space are the main two resources necessary but usually limited for organisms, and they are tightly related to each other. A population colonizing more space will commonly obtain more nutrients and energy to support their growth [10–12]. The space competition of macroscopic organisms has been reported in many papers [2, 12]. However, due to the limitation of the reproduction rate of macroscopic organisms and the size of the competitive space, the sample size in macroscopic ecology research is often relatively small [13], which is not conducive to drawing general conclusions from a wider field of vision (tens of thousands of samples). Microorganisms grow fast and are small in size. Thanks to rapid advances in microscopy and high-throughput sequencing, microbial ecology has developed rapidly, which has also contributed to the understanding of macroscopic communities. For microorganisms, they often settle on surfaces and form a dense biofilm, where nutrient and space are often limited [14], and thus strong microbial competition is always common here.

To win in a game of microbial competition, numerous competitive strategies have been evolved by microorganisms. For example, microbes can gain competitive advantages by privatizing nutrient resources [15, 16], and microbes may obtain fitness benefits by diversifying metabolic mode [17, 18]. In addition, motility [19, 20] and adhesion [21, 22] also help microorganisms win the competition. All the competitive strategies above that these organisms have evolved are biotic factors that affect the outcome of microbial competition.

In addition to the biotic factors, microbial competition is also affected by abiotic factors. Several reports have shown that certain abiotic factors, such as temperature and pH, influence the outcome of microbial competition by changing the intrinsic properties of organisms like growth rates [23, 24]. A recent study has shown that emigration rates, i.e., rates at which individuals of a population depart from a particular community, influence the outcome of microbial competition, without changing the fitness of the competing organisms [25]. However, whether there are other abiotic factors that do not influence the population growth rates but will also affect the outcome of microbial competition, remains to be elucidated. Understanding this question is critical to explain how slow-growing microbes compete against their fast-growing counter-partners and exist in all environments [26–28].

Ecological processes occur not only in time but also in space. Abiotic spatially related factors may also be potential factors independent of biological intrinsic properties but affecting outcomes of the competition for space. Our previous study indicated that even if the initial abundance and inherent fitness of two populations were identical, outcomes of the spatial competition were not completely random but significantly influenced by the relative positions and time orders for the emergence of different genotypes [29]. Similar phenomena have been observed in the macroscopic plant ecology, such as chance seed dispersal and early site history [30], emphasizing the role of temporal order-of-arrival and stochastic processes [31, 32]. These findings suggested that specific events that occurred during the space colonization affected which population colonized more space. However, what these events are and how they affect the outcome of spatial competition, has not been studied enough in previous research, and then more research is needed.

Microbial spatial competition is very similar to the traditional Chinese board game, *Go*, in which two players compete for occupying more space on a board. In the *Go* game, the players’ strategies in layout and middle stage are crucial to gaining more territory and winning the game [33]. Therefore, we hypothesized that factors related to the manner of microorganisms exploring and colonizing free space, such as initial spatial positions and the subsequent directions of expansion, play a significant role in their competitive outcome.

In this study, we constructed an individual-based model (named “BacGo”) to simulate two microbial populations competing for limiting space and explore the influence of spatially related factors on the competition for space. Our work provides a quantitative view of how the manner in which a microbial strain colonizes new space affects the outcome of competing with other strains.

## Methods

### Basic settings and simulation workflows of the BacGo model

**Fig. 1:**
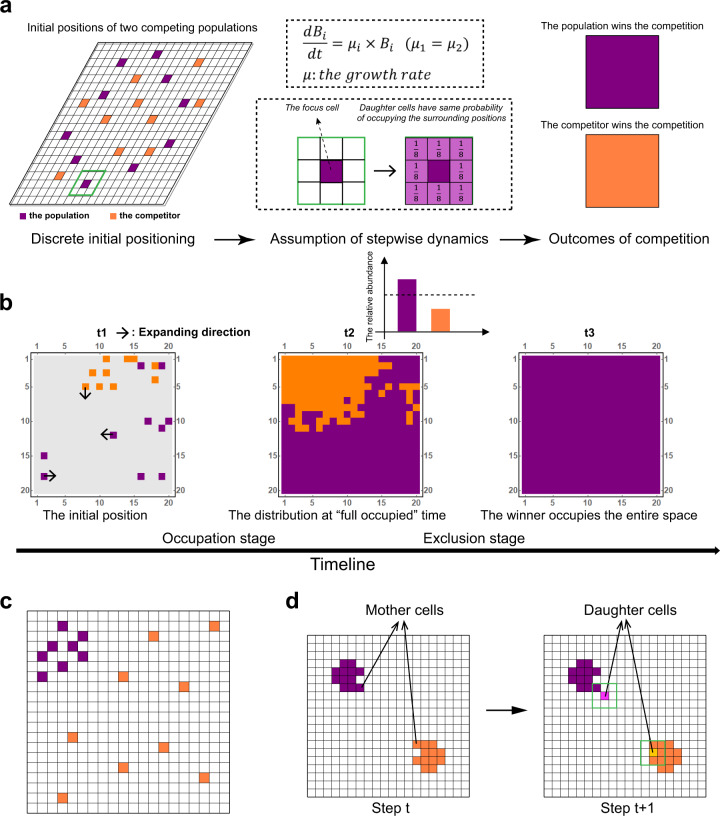
Logic and basic assumptions of the model. **a** Overall framework of the model. We considered two populations competing for a limited 2D space. The space was initialized by a 1:1 mix of cells from two populations, which were randomly scattered in the 2D panel (left panel). Cells from the two populations exhibited the same growth rate. Cell division occurred when its biomass reached a threshold, during which the daughter cell randomly selected one of the directly adjacent grids. If the selected grid had been colonized, the newborn cell competed for the grid with its original occupant and have a 50% probability to survive (middle panel). We aimed to see which population ultimately colonized the entire space (right panel) and for this purpose investigated the relationship between the colonization manner of one population and its competitive success. **b** The process of spatial competition can be divided into two stages, namely the ‘occupation stage’ and the ‘exclusion stage’. Small black arrows indicate the direction of population exploitation; t_1_, t_2_, and t_3_ refer to the initial time point, the “full occupied” time point, and the winner colonizes the entire space time point in the process of competitive interaction, respectively. **c** A representative snapshot shows the difference in the initial distribution between the two populations. The orange population was more scattered than the purple one. **d** Representative snapshot showing the difference in ‘expansion freedom’ between the two populations. Daughter cells (labeled as light purple) of the purple population were characterized by a higher degree of expansion freedom than the daughter cells (labeled as light orange) of the orange population.

To simulate the spatial competition between two populations, the BacGo model was built based on 2D lattices [34], following our previous framework [29]. In our model, a microhabitat was conceptualized as a 20 × 20 array. One microbial individual was allowed to occupy a specific spatial grid box (as shown in the left panel of Fig. 1a). Two populations were assumed to compete for this ‘microhabitat’, where they were allowed to grow and reproduce (the middle panel of Fig. 1a). For simplicity, nutrients are assumed to be unrestricted in the model and the growth rate of each cell was assumed to be constant, then

$$\frac{{dB_i}}{{dt}} = \mu _i \times B_i,$$

where $B_i$ is the biomass of the *i*th individual; $\mu _i$ is the growth rate of the *i*th individual. In the basic model, the $\mu _i$ was set to be the same for all cells of both populations, of which the default value was 0.1 fg/fg∙min [35]. The initial biomass of each individual was set as 150 fg [35]. After enough cycles for biomass accumulation, one cell reproduced when its biomass reached the upper threshold of 2*B*_*0*_. After cell division, the mother cell stayed in the original grid box, while the daughter cell randomly selected one of the 8 (5 when the mother cell is on the edge, and 3 when the mother cell is in the corner) directly adjacent grids. If the selected grid has been occupied, the newborn cell will compete for the grid with its aborigine and have a 50% probability to survive. In addition, random death events were considered and set at a very low probability of 1e^−4^ [36].

According to the basic settings of the model, the competitive processes (simulation workflows) went through two stages (Fig. 1b). In the first stage (named “occupation stage”), cells grew, divided, and occupied the space from initial positions at time point t_1_, until the space was fully occupied by both populations at time point t_2_. In the second stage (named “exclusion stage”), cells from both populations competitively excluded each other, until one population completely filled the entire space (named ‘winner’) at time point t_3_. All variables and parameters used in the model were listed in Table S1, the defined indexes were summarized in Table S2, and the abbreviations were summarized in Table S3.

### Simulation protocols and data recording

To simulate the processes of two populations competing for the ‘checkerboard’, time-lapse numerical simulations lasted for at least 50,000 steps, until one population fully occupied the entire space. The model was implemented by C++ language, and the source code is available on https://github.com/Neina-0830/BacGo-model. Simulations were run on an Ali cloud server running Windows Server 2019. Custom functions were included in the Codes to record the position coordinate of every cell, biomass of every cell, as well as the relative abundance of each population at each time point. These simulation data were analyzed and visualized using custom Wolfram Mathematica scripts (https://github.com/Neina-0830/BacGo-model). Moreover, images of the cell distributions at each time point were generated from custom Wolfram Mathematica scripts (https://github.com/Neina-0830/BacGo-model). Videos were created from the time-series image sequences and assembled in software Image J (v2.35). Videos were reprocessed with H.264 encoding using FormatFactory (v5.7.5) to make sure the suitable size.

### Definition of measurements and parameters

#### Measurements that characterize outcomes of the competition

To statistically characterize the competitive outcome at t_3_, we defined two measurements. In each simulation, the relative abundance of each population at ‘full-occupied’ time point (t_2_) was extracted, and the abundance asymmetry index, AbunR, was calculated as follows:

$${{{{{{{\mathrm{AbunR}}}}}}}} = {{{{{{{\mathrm{log}}}}}}}}\left( {\frac{{Abun_1}}{{Abun_2}}} \right),$$

where $Abun_1$ is the relative abundance of the focus population (the first population) at t_2_ and *Abun*_2_ is the relative abundance of its competitor at t_2_. AbunR of a population >0 means that the focus population had a higher relative abundance than its competitor at t_2_.

When applicable, 100 replicated simulations were performed for one initial cell distribution, and competition outcomes were summarized to get the winning frequency of both populations. Then, the winning asymmetry index, WinR, was calculated as follows:

$${{{{{{{\mathrm{WinR}}}}}}}} = {{{{{{{\mathrm{log}}}}}}}}\left( {\frac{{winpro_1}}{{winpro_2}}} \right),$$

where $winpro_1$ is the winning frequency of the focus population in the 100 replicated simulations starting from the same initial distribution, while $winpro_2$ is the winning frequency of its competitor. When the WinR of a population is positive, this population has a higher winning probability than its competitor.

#### Parameters describing different colonization manners on spatial competition

To investigate the effect of different colonization manners on spatial competition, we defined serval parameters quantifying spatially factors. To characterize the initial population distributions, a nondimensional parameter, ScatR, was defined to assess the asymmetry of scatter level of the initial cell distribution between a population and its competitor, calculated by

$$ScatR = {{{{{{{\mathrm{log}}}}}}}}\left( {\frac{{a_1}}{{a_2}}} \right),$$

where $a_1$ and $a_2$ are the average Euclidean distance of all cells from the average position in the population of two populations respectively (see more details in Supplementary information S1), which characterize the scattered level of initial cell distribution of each population. The ScatR >0 indicates that the population is initially distributed more scattered than its competitor, and the absolute value of ScatR represents the degree of the difference in the scatter level of the initial cell distribution between the two populations.

To capture the random events occurring during population expansion in the “occupation stage”, a parameter FreeR was defined to characterize the difference in the degree of ‘expansion freedom’ between one population and its competitor, given by

$${{{{{{{\mathrm{FreeR}}}}}}}} = {{{{{{{\mathrm{log}}}}}}}}\left( {\frac{{\mathop {\sum }\nolimits_{t = 0}^{t_2} \overline {freedom_{1,t}} }}{{\mathop {\sum }\nolimits_{t = 0}^{t_2} \overline {freedom_{2,t}} }}} \right),$$

where $\overline {freedom_{1,t}}$ is the average number of empty grids around the daughter cells born in time point *t* of the focus population, while $\overline {freedom_{2,t}}$ is that number of its competitor. The FreeR index >0 indicates that the population possesses greater ‘expansion freedom’ against its competitor in the given simulation, and the higher absolute value suggests a higher difference in expansion freedom between the two populations.

In order to integrate the effect of initial cell distribution and ‘expansion freedom’, a new parameter, named ‘Space Accessibility’, was defined. The asymmetry of ‘Space Accessibility’, SAR, evaluated the competitive edge derived from ‘Space Accessibility’ of the population across the whole “occupation stage”, given by

$${{{{{{{\mathrm{SAR}}}}}}}} = {{{{{{{\mathrm{log}}}}}}}}\left( {\frac{{\mathop {\sum }\nolimits_{t = 0}^{t_2} SA_{1,t}}}{{\mathop {\sum }\nolimits_{t = 0}^{t_2} SA_{2,t}}}} \right),$$

where *SA*_1,*t*_ and *SA*_2,*t*_ are the ‘Space Accessibility’ for the focus population and its competitor at time point *t*, respectively. Details of the definition of *SA*_*k*,*t*_ (k = 1 means the focus population and k = 2 means its competitor) can be found in Supplementary information S1. SAR is >0 means that the population generally possesses higher ‘Space Accessibility’ than its competitor.

According to the above definition, the ScatR, FreeR, and SAR of the focus population are all inverse to its competitor, and the competition results of AbunR and WinR are also inverse to each other. Therefore, we can draw a conclusion by focusing only on all the parameters of the focus population, which is completely consistent with the conclusion reached when considering all the parameters of the two populations.

### Comparison between SmartBac and NormalBac

To test the effect of space colonization manners from another perspective, one population was defined to be “smart population” (named as SmartBac thereafter), whose daughter cells were always able to colonize the space to ensure that the whole population retained optimal spatial distribution with higher ‘Space Accessibility’. To achieve this goal, SmartBac was controlled to colonize space in a more dispersed manner. After each daughter cell was born, every possible scenario for its follow-up position was assessed by calculating the *SA*_*k,t*_ value of the formed cell distribution. Then the distribution with maximum *SA*_*k,t*_ value was selected as the next colonizing step of the SmartBac.

To test whether SmartBac behaves better in spatial competition, individual-based simulations were performed by considering a competition process between SmartBac and NormalBac (a normal population), who possessed a purely random manner of colonization of space same as the definition in the basic model. We called the spatial competition between SmartBac and NormalBac the SmartGo. As a control, the corresponding null model simulations, completely random simulations of two populations, were also performed between Normalbac and Normalbac. Finally, we compared parameters of the focus SmartBac in SmartGo with those of the focus NormalBac in the null model to reach a conclusion.

In order to explore the impact of spatial dispersion on competition from a wider perspective, we further defined a parameter ${{{{{{{\mathrm{GrowAdv}}}}}}}}_{NormalBac}$ to reflect the growth rate advantage of NormalBac relative to SmartBac, calculated by

$${{{{{{{\mathrm{GrowAdv}}}}}}}}_{NormalBac} = \frac{{Gro_{NormalBac} - Gro_{SmartBac}}}{{Gro_{smartBac}}},$$

where $Gro_{NormalBac}$ and $Gro_{SmartBac}$ are the growth rate for NormalBac and SmartBac, respectively. We designed simulations of competition between SmartBac with a certain growth rate and NormalBac with different growth advantages, to investigate the advantage of SmartBac in an efficient, dispersed strategy. We also defined a SmartBac attribute, namely the proportion of SmartBac (see Supplementary Information S1 for details), and designed ls of simulations of competition between NormalBac and different proportions of SmartBac to explore competitive outcomes of different intermediate strategies. Finally, we analyzed the outcomes of these simulations.

### Statistical analysis

The chi-square test was carried out using the *chisq.test* function in stats package of R 4.0.2. Unless indicated otherwise, unpaired, two-tailed, two-sample Student’s *t* test was performed for comparative statistics using the *t.test* function in stats package of R 4.0.2. To avoid the impact of the data size on significance analysis, all of the *t*-tests were carried out with 1000 values randomly selected from each group sample. Linear correlation analyses between different parameters were implemented using the *lm* function in stats package of R 4.0.2. The Cohens’D statistic was calculated using the *cohensD* function in lsr package of R 4.0.2. The multiple regression analysis and multicollinearity test were performed using IBM SPSS Statistics 27.0.

## Results

### Simulating competition for space using the “BacGo” model

To investigate how spatial positioning of populations affects the outcome of microbial competition, we simulated two populations competing for space with a limiting size by building an individual-based model (named “BacGo”). The model was implemented in discrete grid boxes of a 20 × 20 array. As shown in Fig. 1a, our simulations were based on three basic assumptions. First, the two competing populations possess the same inherent growth rate and equal initial cell numbers, thus the only differences between them are their manners of colonizing free space. Second, the newly born daughter cell is located around its mother cell but with a random direction of spatial positioning [34], resulted in a microcolony with different spatial patterning. Lastly, if the selected box has been occupied, the newborn cell will compete for the box against the original occupants of the box and possesses a probability of 50% to survive [37].

We first explored the outcome of spatial competition, which started by randomly distributing two populations on the grids with the same initial cell numbers of 10 for each. Based on our basic assumptions and the predictions of competitive exclusion theory [38], we hypothesized that only one population could win the competition and finally occupy all grids. As shown in 20,000 independent simulations with random initial distributions, we discovered that at the end of each simulation, only one population survived (Video S1 and Video S2). The Chi-square test showed no significant difference (*P* = 0.211) between the simulated winning times (10,177 of 20,000 simulations) and the random winning times (10,051 of 20,000 simulations) of the focus population. This result conformed with our initial assumption that cells possess a probability of 50% to survive in competing with original occupants. When we replicated simulations initiated with the same cell distribution, we found that the winning probabilities for each population changed in line with the initial distributions (Fig. S1). However, the winning probabilities never reached 100% no matter how the initial distribution changes. Together, these results suggested that unknown random factors may affect the final outcome of the competition.

Next, we analyzed the dynamics of microbial colonization during our simulations. As summarized in Fig. 1b, we divided the competition process into two stages, the “occupation stage” and the “exclusion stage” (see Methods). To statistically characterize the competitive outcome at t_3_, we defined the winning asymmetry index, WinR, and the abundance asymmetry index, AbunR (see Methods). As shown in Fig. S2a, we found a strong positive correlation (*R*^2^ = 0.740, *P* < 0.001) between AbunR and WinR, indicating that if any population is more abundant at the “full occupied” time (t_2_), it is more likely to finally win the competition (i.e., occupy the entire space at t_3_) (Fig. S2b). These results strongly suggested that one population may obtain an asymmetric benefit from the random manners of colonizing space in the “occupation stage”, a benefit that assists this population in colonizing more space at t_2_, thus largely determining the ultimate outcome of the competition.

All of these initial explorations of the model indicated that, in addition to the growth rate [39] and initial cell numbers [40], the random manners of colonizing space in the “occupation stage” may provide a considerable competitive edge for a population to colonize space.

### ‘Space Accessibility’ affects outcomes of spatial competition

#### A larger initial distance of cells is conducive to success in competition

We next investigated that what manners of colonizing space will help to win the competition. Since the competition outcome changes with different initial cell distributions (Fig. S1), we first explored how the differences in features of initial cell distributions affect the outcome of subsequent spatial competition. Our model assumed that the direct competition between different cells occurred only when cells are located adjacent to each other (assumption 2 and assumption 3). Based on these assumptions, if cells from one population possess greater distance among each other (in other words, distributed more scattered), the undesirable intrapopulation competition can be avoided, and thus they may possess a higher probability to occupy more space. Therefore, we hypothesized that if one population exhibited a higher degree of scatter at time point t_1_, it will potentially occupy more space at time point t_2_, resulting in a higher probability to emerge as the winner.

**Fig. 2:**
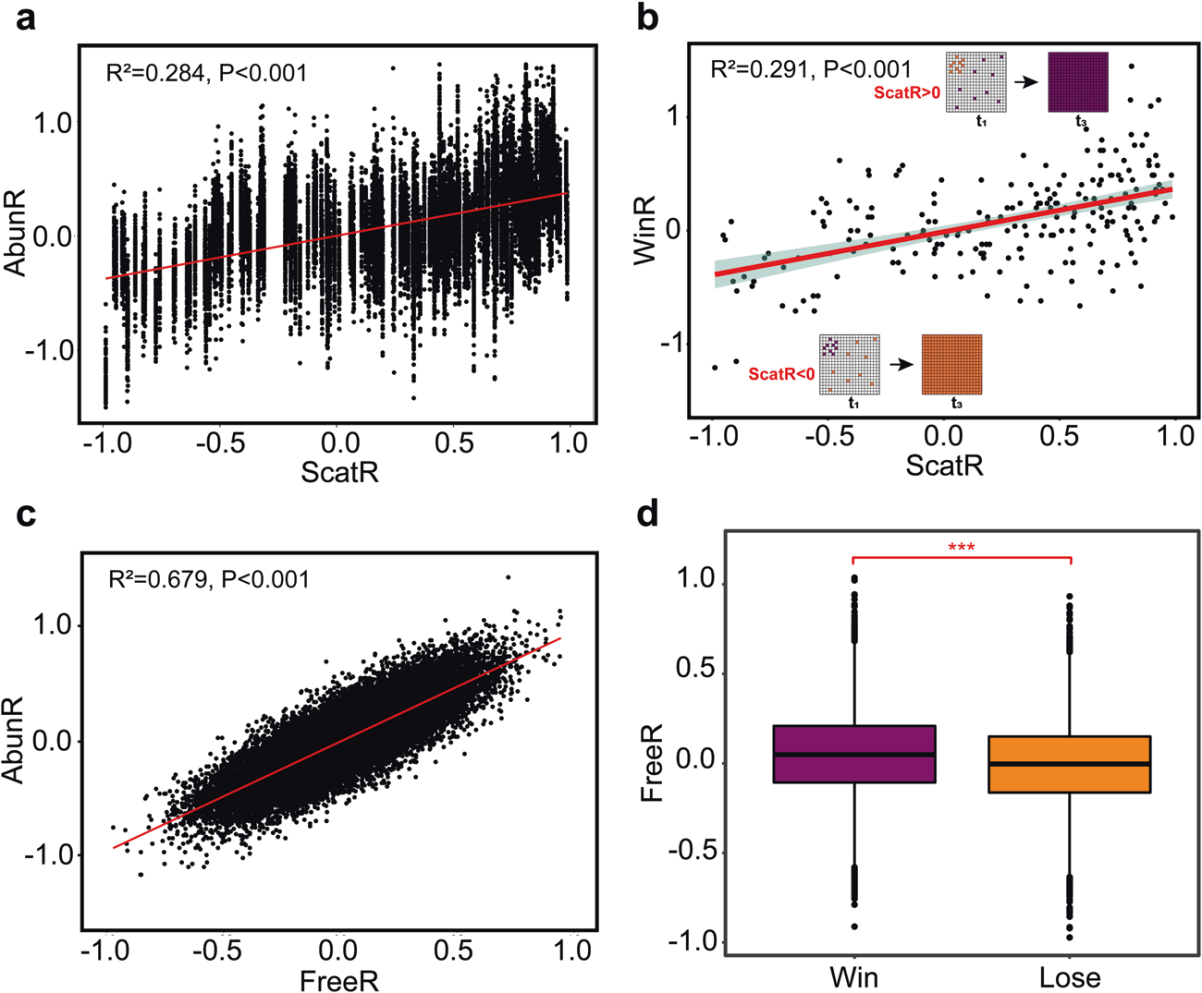
Effect of the initial cell distribution and expanding direction of the daughter cells on competitive success of one population. **a** Correlation between ScatR and AbunR. Results represented the sum of 21,500 simulations containing 215 different initial cell distributions, of which the ScaR values of the focus population varied from −1.009 to 1.053. **b** Correlation between ScatR and WinR. Data were generated from simulations identical to those shown in (**a**), and each WinR value was summarized from the competition outcome of 100 replicated simulations with a given initial cell distribution. **c** Correlation between FreeR and AbunR. Results represented the sum of 36,300 independent simulations with 363 different initial cell distributions, but their ScaR values were all equal to zero (Fig. S3). **d** Comparison of FreeR in the winning and losing cases of the focus population. Competition outcomes were generated from simulations identical to those shown in (**c**). Statistical analysis was performed using a two-sample Student’s *t* test: ***p < 0.001.

To compare levels of scatter (Fig. 1c) of the initial cell distribution between two populations, we defined the scatter asymmetry index, ScatR (see Methods). To investigate whether the initial scatter level affects the competition outcome, we selected 215 initial cell distributions randomly, which covered a gradient of ScatR values of the focus population ranging from –1.009 to 1.053 (Blue lines in Fig. S3). We then performed 100 replicated simulations for each initial distribution, to reveal the relationship between the competition outcome and ScatR. Our results showed that AbunR was positively associated with ScatR at significant levels (Fig. 2a; *R*^2^ = 0.284, *P* < 0.001), indicating that the population initialized with more scattered cell distribution would occupy more space at t_2_. Moreover, a positive relationship was also observed between WinR and ScatR (Fig. 2b; *R*^2^ = 0.291, *P* < 0.001), further suggesting that the benefit obtained from more scattered initial cell distribution contributed to the ultimate dominance of this population.

#### The higher degree of expansion freedom helps populations to win space competition

In addition to initial distance, we found that for a given initial distribution, AbunR considerably varied across different expansion processes, indicating that in addition to the randomness in the initial cell distribution, the random events occurring during population expansion in the “occupation stage” also affected the competition outcome. Our model assumed that after a successful division of one cell, all adjacent grids around the mother cell are randomly selected to accommodate the newly born cell (Fig. 1a; assumption 2). If the selected box has been occupied, the newborn cell will compete for the box with the aborigine of the box and has a 50% probability to survive (assumption 3). We defined the number of empty grids surrounding the newborn cell as the degree of expansion freedom. Thus, if the daughter cell possesses a higher degree of expansion freedom, the probability for its offspring to survive will be higher (Fig. 1d; the purple cell). In contrast, if the degree of expansion freedom of the daughter cell is low, it has to compete for space with other cells for further reproduction and expansion, which should be less favored for the space competition afterward (Fig. 1d; the orange cell). This assumption leads to a prediction that the population whose daughter cells possess a higher degree of expansion freedom will be more likely to win the competition.

To test this prediction, we defined the expansion freedom asymmetry index, FreeR (see Methods). We selected 363 initial cell distributions with a zero ScatR (Fig. S3) from 1,000,000 random distributions and performed 100 replicated simulations with each initial distribution. During these simulations, we recorded the degrees of expansion freedom of every newborn cell during the ‘occupation stage’ (Fig. 1d; Fig. S4) and then compared the FreeR of the focus population with its AbunR in each simulation. We observed a strong positive relationship between FreeR and AbunR (Fig. 2c; *R*^2^ = 0.679, *P* < 0.001), suggesting that the population with greater ‘expansion freedom’ would occupy more space at t_2_. Furthermore, the FreeR of the focus population was significantly higher when it won than it lost (Fig. 2d; *t*-value = 5.343, df = 999, *P* < 0.001), which is consistent with our prediction.

Together, these results demonstrated that the randomness during the “occupation stage” of spatial competition, including the initial scatter level and the degree of expansion freedom, can affect the outcome of competition for space.

#### Populations with higher ‘Space Accessibility’ have a higher winning probability in spatial competition

Because both the initial scatter level and the degree of expansion freedom affect the number of empty grids that surrounded individuals of the focus population at each time point, we then searched for a more general parameter that considered both of the two factors. We applied a mathematical induction algorithm to define a new parameter, Space Accessibility (SA_k,t_, k = 1 means the focus population and k = 2 means its competitor; Fig. S5). Individuals of a population are further away from the aggregation area, where more grids have been occupied, the ‘Space Accessibility’ of this population is higher. The ‘Space Accessibility’ at each time point (SA_k,t_) assesses the maximum probability of cells of one population colonizing all the empty grids in the subsequent steps from this time point to the “full occupied” time (t_2_), which reflects the ease with which offspring cells occupy these empty positions. Next, we integrated SA_k,t_ value over time (obtaining SA) for each population. To estimate which population generally was more likely to occupy the empty positions during the “occupation stage”, we next defined an index called SAR (see Methods). A SAR index greater than zero indicates that the focus population has a higher probability of reaching empty positions than its competitor across the “occupation stage”.

**Fig. 3:**
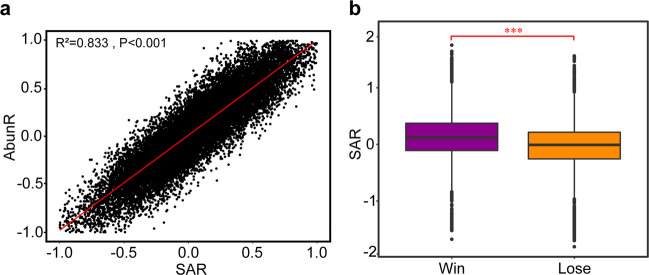
Effect of the ‘Space Accessibility’ on the competitive success of a population. **a** Correlation between SAR and AbunR. Results were summarized in 20,000 independent simulations. **b** Comparison of SAR in the winning and losing cases of the focus population Competition outcomes were generated from simulations identical to those shown in (**a**). Statistical analysis was performed using a two-sample Student’s *t* test: ***p < 0.001.

To investigate whether the difference in ‘Space Accessibility’ affects the competition outcome, we performed 20,000 simulations covering SAR values of the focus population ranging from −1.808 to 1.754. In these simulations, we found that ScatR (Fig. S6a; *R*^2^ = 0.271, *P* < 0.001), as well as FreeR (Fig. S6b; *R*^2^ = 0.986, *P* < 0.001), was positively correlated with the SAR, suggesting that SAR reflected the change of both ScatR and FreeR. To test the influence of ‘Space Accessibility’ for competition, we next analyzed the relationship between SAR of the focus population and its AbunR at t_2_ time point. The results showed an extremely significant positive correlation between SAR and AbunR (Fig. 3a; *R*^2^ = 0.833, *P* < 0.001), suggesting that the population with higher ‘Space Accessibility’ would occupy more space at t_2_. The correlation coefficient between AbunR and SAR was higher than the coefficients of both AbunR-ScatR and AbunR-FreeR, indicating that SAR represented a more suitable parameter to evaluate competition outcomes. Furthermore, the SAR of the focus population was significantly higher when it won than it lost (Fig. 3b; *t*-value = 8.392, df = 999, *P* < 0.001), further indicating that the ‘Space Accessibility’ predicted the outcome of spatial competition between two populations with a high degree of reliability.

We also performed numerous well-designed simulations (see Supplementary Information S2 for details) to test whether the effect of ‘Space Accessibility’ on the outcome of spatial competition was statistically significant under various initial conditions (robustness test), including varied initial growth rates, total numbers of initial cells, as well as sizes of the space (Table S4). Our analysis showed that the effect of ‘Space Accessibility’ on the outcome of spatial competition was significant (Table S4; Fig. S7), and largely unperturbed by changes in initial growth rates, space sizes, and initial total numbers of cells (not exceed 10% of the maximum population size). In summary, colonizing space in a more dispersed manner contributes to microbial competitive success.

#### A ‘smart population’ occupies more space

Next, we tested whether ‘Space Accessibility’ determined the competition outcome from another perspective. We designed simulations of competition between SmartBac and NormalBac (called ‘SmartGo’; see Methods). We hypothesized that SmartBac would obtain a higher competitive edge from its superior strategy of space colonization, and win the competition for space against NormalBac.

**Fig. 4:**
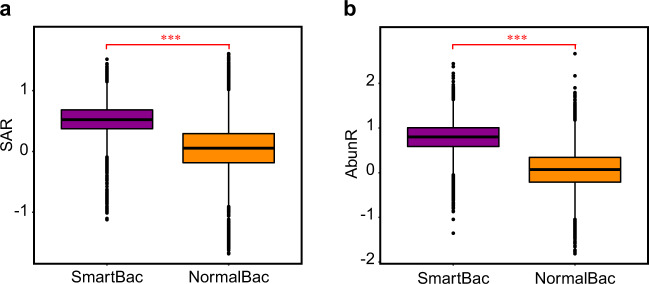
Microbial colonization of space in a more dispersed manner contributes to the competitive success of the SmartBac. **a** Comparison of the SAR of SmartBac in SmartGo and the focus NormalBac in the null model in the “occupation stage”. **b** Comparison of the AbunR at t_2_ of SmartBac in SmartGo and the focus NormalBac in the null model. Results were summarized in 10,000 independent simulations of SmartBac competing with NormalBac and 10,000 independent simulations of NormalBac competing with NormalBac. Statistical analysis was performed by a two-sample Student’s *t* test: ***P < 0.001.

As expected, SmartBac won (Video S3) 7302 times during 10,000 mathematical simulations of the SmartGo model, accounting for 73.02%. While, in the corresponding null model (competition between NormalBac and NormalBac), the focus NormalBac won 5088 times during 10,000 mathematical simulations, accounting for 50.88%. In these 20,000 simulations, SAR values of the SmartBac in the SmartGo model were significantly higher than those of the focus NormalBac in the null model (Fig. 4a; *t*-value = 30.104, df = 999, *P* < 0.001). Furthermore, the AbunR values of SmartBac in SmartGo model were also significantly higher than those of focus NormalBac in null model (Fig. 4b; *t*-value = 40.763, df = 999, *P* < 0.001).

We also designed simulations of competition between SmartBac and NormalBac, and the latter has a growth advantage. A parameter ${{{{{{{\mathrm{GrowAdv}}}}}}}}_{NormalBac}$ was defined to reflect the growth rate advantage of NormalBac relative to SmartBac (see Methods). The results showed that as the ${{{{{{{\mathrm{GrowAdv}}}}}}}}_{NormalBac}$ increased, the winning probability of SmartBac decreased (Fig. S8). The winning probability curve of SmartBac intersects the line of 50% winning probability at (0.0083, 0.5), which means that SmartBac can compete with NormalBac, which has a 0.83% growth advantage, by compensating for the growth disadvantage with a more dispersed strategy.

In order to explore the impact of ‘Space Accessibility’ on competition from a wider perspective, we further defined an attribute for SmartBac, namely the proportion of SmartBac (see Methods). We ran *60,000* simulations of competition in total between NormalBac and different proportions of SmartBac (see Supplementary Information S1). The results showed that populations with higher proportions of SmartBac had average higher SAR values across all competitive simulations (Fig. S9a). Moreover, the population with a higher proportion of SmartBac had a higher probability of winning in the competition with Normalbac (Fig. S9b).

Together, the results further indicated that microbial colonization of space in a more dispersed manner helped to win the competition.

### Space colonization manners, growth rates, and initial abundances synergistically affect spatial competition

**Fig. 5:**
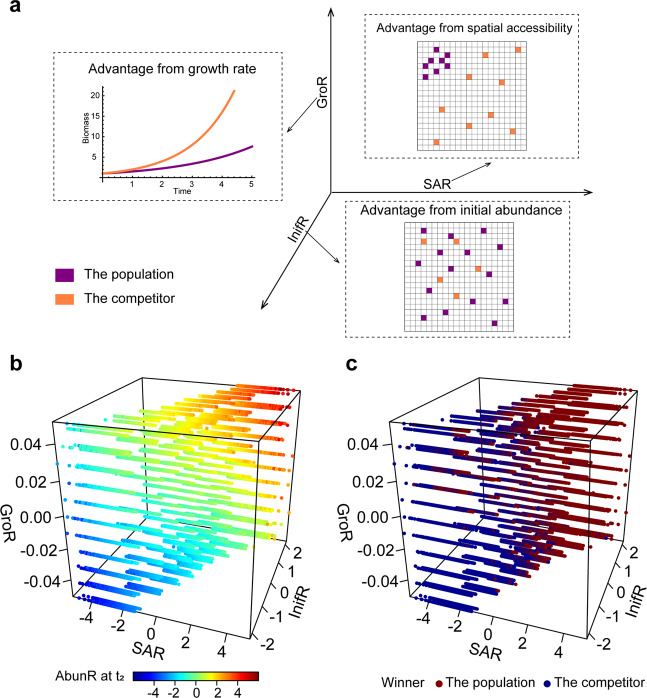
Comparison of the effect of space colonization manners with the effect of the varied growth rate and initial abundance. **a** Diagram indicating meanings of the defined three parameters. Values of each parameter >0 denote that the focus population possesses the corresponding competitive edge compared with its competitor. **b**–**c** Comparison of the relative importance of space colonization manners, growth rates, and initial abundances for outcomes of the microbial competition. Gradients of SAR, GroR, as well as InifR, were set in 89,100 simulations and each point indicated one simulation result in the corresponding set of the three parameters. Values of AbunR (**b**), as well as final competition outcomes (**c**), were also recorded to estimate how these three factors collectively affect microbial competition.

It is well established that microbial competition for space is influenced by the growth rate and initial abundance of competing populations. The population possessing a faster growth rate, or higher initial abundance will outcompete other strains present within the newly occupied space. To assess the relative contribution of space colonization manners, growth rates, and initial abundances in spatial competition, to microbial competitive success, we performed simulations in which the growth rates and initial abundances of the two populations were set to be different (see Supplementary Information S1 for details). In these simulations, we defined the parameter GroR as the difference in growth rate between a population and its competitor, as well as defined InifR to characterize the difference in initial abundances (see Supplementary Information S1; Fig. 5a). In addition, we calculated SAR of the focus population in each simulation to quantify the asymmetry of ‘Space Accessibility’.

As shown in Fig. 5, even when one population exhibited a lower growth rate, or was characterized by the lower initial abundance, colonization of space in a more dispersed manner, such as choosing positions for new cells to have a higher ‘Space Accessibility’, may neutralize these disadvantages and allow this population to occupy more space at t_2_ (Fig. 5b), thus winning the spatial competition (Fig. 5c). To more clearly display comparison results between the spatial advantage obtained by the dispersed strategy and the growth rate or initial abundance advantage, we added two-dimensional plots of GroR and SAR, InifR and SAR in Fig. S10.

The collinearity analysis showed that when strains differed in their initial abundances, SAR and InifR exhibited significant collinearity (VIF = 13.062, VIF is short for variance inflation factor). To eliminate this collinearity effect, we generalized our definition of SAR by defining a new parameter perSAR (see Supplementary Information S1), which is equal to SAR when the initial cell number of both populations are same (InifR = 0), but allows for better quantification of the asymmetry of ‘Space Accessibility’ when InifR is unequal to zero. A subsequent collinearity test showed that the collinearity among the variables perSAR, GroR, and InifR disappeared (Table S5). Moreover, the population possessing a higher perSAR value was characterized by a higher probability for ultimate survival at the end of the simulation and won the competition even when its growth rate or initial abundance was lower (Fig. S11).

We next performed multiple regression analysis to quantify the relative contributions of these three factors during the spatial competition (Table S5). Our analysis showed that the ratio of relative contributions of perSAR, GroR, and InifR to AbunR was ~1.027, 55.393, and 1.027 (1:53.94:1), suggesting that the competitive disadvantage derived from lower GroR of a population could be eliminated by possessing 53.94 times higher perSAR, and the competitive disadvantage derived from lower InifR could be neutralized by 1-time higher perSAR. Together, these results indicated that microbial colonization of space in a more dispersed manner could benefit the competitive success of slow-growing species or species possessing lower seeding abundance.

In summary, compared with the evident competitive edge derived from a faster growth rate and higher initial abundance of one competitor, colonization of space in a more dispersed manner (e.g., possessing higher ‘Space Accessibility’) also played a critical role in determining the success rate during the competition for space between microbial strains.

## Discussion

In this study, we investigated whether and how space colonization manners affected the outcome of spatial competition among different microbial populations. Our results suggested that populations that optimize dispersal by colonizing space in a more dispersed manner, could win the spatial competition even if they grow slightly slower than their competitors.

We considered exploring the spatially related abiotic factors of spatial competition, which were inspired by a traditional Chinese board game, *Go*. In the game *Go*, two players need to rationally consider the strategy of how to place their game pieces in order (strategically determines where to place a piece at each step [33]) to win the game by occupying more area on the board. This process is in close analogy to the ecological processes that two biological populations competitively colonize an uninhabited space. Inspired by the golden rule [33] of winning a *Go* game, “golden corner, silver side, and grass belly”, we proposed the hypothesis that manners of organisms colonizing free space play a significant role in their competitive outcome.

The more dispersed manner, which means preference to expansion into empty position, is interesting but hardly surprising. There are more space resources around the microorganisms in the empty position, and the probability of direct competition is greatly reduced. Like microbes migrating to a new feeding ground, they can grow and reproduce at full capacity until other competitors move in and competition starts. Furthermore, our competition simulation occurs between two populations, which is simple but reliable. Pairwise interaction is the basis of multi-species interaction, and it is very important to study the competitive interaction between two populations to reveal the rules of multi-species interaction [41]. Microcosm experiments have found that individual pairwise interactions are often consistent across community complexity [42, 43]. Similar methods for summarizing the prediction framework using paired interactions and extending it to multi-species communities have been reported in other literature [44, 45]. However, higher-order interactions are also important in the ecosystem [46, 47], and sometimes the community theory of higher-order interactions is contrary to the classic theory that is based on pairwise interactions [48]. Thus, the model of spatial competition between two species is not enough. We will further develop and refine this model in the direction of multi-species or higher-order interactions in the future.

Initial spatial pattern and random processes during microbial population expansion are important for spatial competition [7, 49]. For example, one recent study explored how an *Escherichia coli* population colonized the surface of a flat agarose pad and investigated how two *E. coli* populations compete for limited space [7]. In that study, cells with smaller initial patches are more likely to be winners, which agrees with our model prediction, as more scattered initial distribution normally leads to smaller patches at the beginning of colonization. In addition, random processes such as spatial wandering of so-called ‘pioneers’ at the expanding frontier of a colony [49, 50], will determine the spatial competition among the founder cells. Our findings presented here suggest that the direction toward which the newly divided cells migrate represents an important random event in the expansion of a colony, critically affecting the spatial competition between different populations. The populations generating offspring cells with greater ‘expansion freedom’ will occupy more space at t_2_ and thus gaining an advantage over other strains competing for space. Therefore, space colonization is a vital stochastic factor that governs the interactions between competing microbes, as well as the structure of their communities.

Ecological competition can result in the evolution of phenotypes. Several studies using experimental evolution have documented evolution in spatial competition. For example, a mutant repeatedly arose in a biofilm formed by *Pseudomonas fluorescens* Pf0-1, able to maintain a presence at the surface of the biofilm, thus gaining access to limiting nutrients and space [51]. Here, we hypothesized that the evolution of colonizing space in a more dispersed manner may benefit the spatial competition of microbes. Our simulations indicated that an evolved population (SmartBac), that always migrated to the grids with higher ‘Space Accessibility’, would be selectively favored (Fig. 4). Recent studies have provided clues supporting this evolution strategy. Quorum sensing (QS), a signaling system that regulates gene expression and coordinates population behavior in response to changes in population density, is very common among microbes [52–55]. QS signals can be used to detect the free areas, that the free area is larger, the concentration of QS signals should be lower. The slime mold *Physarum polycephalum* can navigate complex labyrinths to find the optimal path to a food source [56]. In these cases, microorganisms can sense the population density and migrate in a directional manner, suggesting the possibility of the evolution for colonizing space in a more dispersed manner. To test this hypothesis, a long-term experimental evolution assay performing spatial competition of two populations at the individual level should be designed.

The surface colonization processes of bacteria are very common in nature [57]. Several previous studies have shown that the aggregation of cells on surfaces helps to resist environmental stress such as desiccation, antibiotics, and predation [58–60]. One study combining the cell-tracking technique and computer simulations showed that *P.* *aeruginosa* deposits a trail of Psl as it moves on a surface, which leads to aggregation of cells in a rich-get-richer process [61]. In harsh environments, surface-attachment of planktonic cells was shown to be biased toward lower distances to previously attached cells [62]. In our model, however, we did not consider the presence of any other environmental stress except the limitation of space size. In this ideal scenario, we got a completely different conclusion, that the “dispersed” strategy helps the population gain an advantage in the spatial competition, which helps to extend the current ecological theory. A previous study demonstrated that two *V. cyclitrophicus* populations have different ecological strategies to interact with microscale nutrient patches in the ocean [63]. The L population is better at accessing localized resources at the individual patch level and the S population is better at dispersing and discovering new patches. This ecological competition-dispersal tradeoff provides a simple explanation for co-existence [63]. It is also interesting to determine trade-offs between the environmental stress resistance and microbial competition. Further work should combine the effects of “dispersed” behaviors in the competition for space and the “aggregation” behaviors resisting the environmental stresses, to reveal the contributions of competition and environmental adaptation in surface colonization quantitively.

Previous studies have shown that predictions made for macro-organisms at least partly also apply to microorganisms [64]. Although we built our model based on a set of assumptions considering the lifestyle of microorganisms, similar assumptions have been used in models of plant ecology [65]. Thus, specific rules derived from the simulations may also have implications for understanding the space colonization of macroorganisms. Data of 54 natural forest areas from ForestGEO (https://forestgeo.si.edu/) confirms that seeding with more scattered initial distribution contributes to faster space colonization of trees, which is consistent with the conclusion of our microbial competition model (Fig. S12). Therefore, our findings can also be generalized to explain how multicellular individuals compete for space and may help to design ecological restoration strategies, such as artificial forestation.

Our analysis of spatial competition of two populations indicated that the competitive disadvantage derived from a slower growth rate could be neutralized by higher ‘Space Accessibility’. As a result, colonizing space in a more dispersed manner will benefit the competitive success of a slower-growing species. This result provided a novel perspective that a smarter manner for space colonization may contribute to the survival of those slow-growing species. Spatial-structured environments, such as biofilm or soil, commonly exhibit higher spatial heterogeneity [66–69], characterized by numerous homogeneous microhabitats [70]. The competition outcome in each microhabitat varies due to random space colonization, allowing the co-existence of species with different growth rates at a large spatial scale. Therefore, our results also provide novel insights into the maintenance of biodiversity in spatial-structured environments.

Our results clearly demonstrate that disadvantaged strains can use innovative strategies when colonizing newly discovered space, compensating for disadvantageous biotic conditions, and thus considerably improving its changes in the evolutionary arms race. These findings shed light on the role of spatial positioning in maintaining biodiversity within natural communities, as well as provide new insight on how spatial competition between different populations drives evolutionary innovation.

## Supplementary information

Supplementary Information

Video S1

Video S2

Video S3
