# Supplementary material for: Successful microbial colonization of space in a more dispersed manner
Source: ISME Commun. 2021 Nov 20;1:68. doi: 10.1038/s43705-021-00063-7 (PMC9723722; doi:10.1038/s43705-021-00063-7)
Supplement: Supplementary file 1 — Supplementary Information [file 43705_2021_63_MOESM1_ESM.docx]

**SUPPLEMENTARY INFORMATION**

**for**

**Successful microbial colonization of space in a more dispersed manner**

Xiaonan Liu^1^, Miaoxiao Wang^1^, Yong Nie^1*^, and Xiao-Lei Wu^1, 2, 3*^

^1^ College of Engineering, Peking University, Beijing 100871, China

^2^ Institute of Ocean Research, Peking University, Beijing 100871, China

^3^ Institute of Ecology, Peking University, Beijing 100871, China

^*^Corresponding author: Research Scientist, College of Engineering, Peking University.

Tel: +86 10-62759047; Fax: +86 10-62759047; E-mail: nieyong@pku.edu.cn

^*^Corresponding author: Professor, College of Engineering, Peking University.

Tel: +86 10-62759047; Fax: +86 10-62759047; E-mail: xiaolei_wu@pku.edu.cn

**S1 Simulations designed for investigating the effect of different colonization manners on spatial competition**

**Simulations for investigating the effect of initial cell distribution on spatial competition**

Firstly, a non-dimensional parameter, ScatR, was defined to assess the asymmetry of scatter level of the initial cell distribution between a population and its competitor, calculated by:

$$a_{1}=\frac{\sum_{i=1}^{n_{1}} \sqrt{{(x_{1i}-\bar{x_{1}})}^{2}+{(y_{1i}-\bar{y_{1}})}^{2}}}{n_{1}}$$

$$a_{2}=\frac{\sum_{i=1}^{n_{2}} \sqrt{{(x_{2i}-\bar{x_{2}})}^{2}+{(y_{2i}-\bar{y_{2}})}^{2}}}{n_{2}}$$

$$ScatR=log(\frac{a_{1}}{a_{2}})$$

In which, $(x_{1i}, y_{1i})$represents the position coordinate of the *i*th individual of the focus population, while $(x_{2i}, y_{2i})$represents that of its competitor. $n_{1}$and $n_{2}$ are initial cell numbers of the two populations, respectively. $a_{1}$ and $a_{2}$ are the average Euclidean distance of all cells from the average position in the population of two populations respectively, which characterize scattered levels of initial cell distribution of the two populations. Therefore, ScatR reflects the scatter asymmetry of the focus population in the initial distribution. The ScatR greater than 0 indicates that the population is initially distributed more scattered than its competitor, and the absolute value of ScatR represents the degree of difference in scatter level of the initial cell distribution between the two populations.

Secondly, to obtain initial cell distributions with different relative scattered levels, 1000000 cell distributions were randomly generated, and ScatR values of these distributions were calculated (Fig. S3). According to these calculations, 215 distributions were selected, which covers a gradient of ScatR values of the focus population ranged from -1.009 to 1.053 (in other words, when ScatR increased by 0.01, approximately one initial distribution was selected).

Thirdly, 100 replicated simulations were performed initialized with each cell distribution and 21500 simulations in total were run. AbunR and WinR values of these simulations then were calculated and linear correlations between ScatR, AbunR, and WinR, were analyzed.

**Simulations for investigating the effect of ‘expansion freedom’ on spatial competition**

In order to investigate the effect of degree of ‘expansion freedom’ on the competition outcome, 363 cell distributions were selected from the 1000000 ones generated from the above part, of which the ScatR values were all equal to zero (Red line in Fig. S3). Also, 100 replicated simulations were performed from each initial distribution, and 36300 simulations were performed in total. During simulations, the expanding direction (position coordinates) of every offspring cell was tracked in detail. After simulations, a parameter FreeR was defined to characterize the difference in the degree of ‘expansion freedom’ between one population and its competitor, given by

$FreeR=log(\frac{\sum_{t=0}^{t_{2}} \bar{{freedom}_{1,t}}}{\sum_{t=0}^{t_{2}} \bar{{freedom}_{2,t}}})$

Here, the $\bar{{freedom}_{1,t}}$ is the average number of empty grids around offspring cells born in time point$t$ of the focus population, while $\bar{{freedom}_{2,t}}$ is that number of its competitor. The summation of $\bar{freedom}$across the “occupation stage” reflects average empty-position numbers surrounding the population during the spatial competition. Here, the FreeR index greater than zero indicates that the focus population possesses greater ‘expansion freedom’ against its competitor in the given simulation, and the higher absolute value suggests a higher difference in expansion freedom between the two populations. Based on this definition, FreeR evaluates the competitive edge derived from the asymmetric ‘expansion freedom’ of one population against its competitor across the “occupation stage”.

Finally, linear correlations between AbunR and FreeR of each simulation, and differences of FreeR values between simulations when the focus population won and lost, were statistically analyzed.

**Simulations for investigating the effect of ‘Space Accessibility’ on spatial competition**

In order to integrate the effect of initial cell distribution and ‘expansion freedom’, a new parameter, named ‘Space Accessibility’, was defined. Firstly, ${SA}_{k,j,t}$ was defined as the maximum probability that offspring cells of the *j*th individual of the *k*th population occupy all unoccupied grids from time point t to the ‘full-occupied’ time point (t_2_). To calculate ${SA}_{k,j,t}$, all unoccupied grids were divided into 19 layers centered with the grid of *j*th individual (since the whole space was a 20×20 array, and there are at most 19 neighbor layers around the *j*th individual; Fig. S5). As shown in Fig. S5, according to mathematical induction methods, the total numbers of shortest paths from the position of *j*th individual to grids in the *i*th layer, $N_{i, j}$, was calculated by

$N_{i,j}=8\times\sum_{m=0}^{i-1} 3^{m}$,

where m is the cyclic variable in the formula for calculating the sum. Along each path, the maximum probability of offspring cells of the *j*th individual to occupy a grid in the *i*th layer, $P_{i, j}$, was calculated by

$$P_{i,j}={(\frac{1}{8})}^{i}$$

Next,${SA}_{k,j,t}$ was derived by

$${SA}_{k,j,t}=\sum_{i=1}^{19} {N_{i,j}\times P}_{i,j}\times G_{k,i,j,t}$$

Here, $G_{k,i,j,t}$ is the number of empty grids in the *i*th layer surrounding the *j*th individual of the *k*th population at time point *t*. To assess the maximum probability of cells of the *k*th population occupy all the empty space at time point *t* in the follow-up steps, ‘Space Accessibility’ of the *k*th population was then defined as the summation of ${SA}_{k,j,t}$ of every individual of the *k*th population at time point *t*, given by

$${SA}_{k,t}=\sum_{j=1}^{n_{k,t}} {SA}_{k,j,t}$$

Here, $n_{k,t}$ is the number of individuals of the *k*th population at time point *t*. Finally, the asymmetry of ‘Space Accessibility’, SAR, was defined to collectively evaluated the competitive edge derived from ‘Space Accessibility’ of the population across the whole “occupation stage”, given by

$$SAR=log(\frac{\sum_{t=0}^{t_{2}} {SA}_{1,t}}{\sum_{t=0}^{t_{2}} {SA}_{2,t}})$$

Here, ${SA}_{1,t}$ and ${SA}_{2,t}$ are the ‘Space Accessibility’ for the focus population and its competitor at time point t, respectively. By this definition, SAR greater than 0 means that the focus population generally possesses higher ‘Space Accessibility’ than its competitor.

To investigate how the ‘Space Accessibility’ affects the outcome of spatial competition between two populations, 200 initial cell distributions were selected, which covered a gradient of ScatR values of the focus population ranged from -1.0 to 1.0. 100 replicated simulations were performed initialized with each cell distribution and 20000 simulations in total were run. In each simulation step, ${SA}_{1,t}$ and ${SA}_{2,t}$ were calculated by the above methods, implemented by custom C++ code (<https://github.com/Neina-0830/BacGo-model>). After simulations, SAR, AbunR, as well as WinR of the focus population in these simulations were calculated and analyzed.

**Simulations for investigating the advantage of SmartBac in a more dispersed strategy on spatial competition**

In order to investigate the advantage of SmartBac in a more dispersed strategy, we designed simulations of competition between SmartBac and NormalBac with different growth rates. In these simulations, the growth rate of SmartBac was set to 0.1 fg/fg∙min, the same as in the base model. However, the growth rate of NormalBac was slightly higher than that of SmartBac, set to 0.1005, 0.101, 0.1015, or 0.102 (the unit is fg/fg∙min). For each set of growth rates, 30 initial cell distributions were randomly selected, and 100 replicated simulations were performed from each initial cell distribution, then 3000 simulations in total were run. These simulations were implemented using modified C++ codes, which are available online on <https://github.com/Neina-0830/BacGo-model>. After all simulations, the winning probabilities of SmartBac were calculated and analyzed.

**Simulations for investigating the effect of proportions of SmartBac on spatial competition**

In order to explore the impact of spatial dispersion on competition from a wider perspective, we further define a SmartBac attribute, namely the proportion of SmartBac. For example, the 20% proportion of SmartBac means that in the SmartBac population, 20% of individuals will be completely smart and their offspring cells will occupy the neighboring grids with maximum ‘Space Accessibility’, while the remaining 80% will have their offspring cells randomly occupy the neighbor grids.

To investigate the effect of proportions of SmartBac on spatial competition, we performed competitive simulations between populations with different proportions (0%, 20%, 40%, 60%, 80%, and 100%) of SmarBac and NormalBac, respectively. For each competition model, 100 initial cell distributions were randomly selected, and 100 replicated simulations were performed from each initial cell distribution, then 10000 simulations in total were run. These simulations were implemented using modified C++ codes, which are available online on <https://github.com/Neina-0830/BacGo-model>. After simulations, SAR values and winning probabilities of the focus population, having partial SmartBac, were calculated and correlations between SAR, winning probabilities, and proportions of SmartBac were analyzed.

**Simulations for comparing the effect of space colonization manners, growth rates, and initial abundances on spatial competition**

To compare the relative importance of the effect of space colonization manners, different growth rates, and different initial abundances for microbial competition, three parameters were defined to assess different asymmetries of the focus population in the above three aspects. Specifically, GroR was defined to assess the asymmetry of the growth rate of the focus population, given by

$$GroR=log(\frac{{Gro}_{1}}{{Gro}_{2}})$$

Here, ${Gro}_{1}$ and ${Gro}_{2}$ are the growth rate of the focus population and its competitor, respectively. Moreover, InifR was introduced to assess the asymmetry of the initial abundance of the population, given by

$$InifR=log(\frac{{Inif}_{1}}{{Inif}_{2}})$$

In which, ${Inif}_{1}$ and ${Inif}_{2}$ are initial cell numbers of the focus population and its competitor, respectively. SAR was used to evaluate the asymmetry of the manner of colonizing space, and it was calculated as described before. Based on these definitions, the values of each parameter greater than 0 denote that the focus population possesses the corresponding competitive edge.

Next, a parameter set containing 89100 combinations of GroR and InifR was designed, in which values of GroR of the focus population varied from -0.0513 to 0.0513, and values of InifR of the focus population were ranged from -2.197 to 2.197. To generate different SAR values for the focus population, 30 different initial cell distributions were established, which covered a gradient of ScatR values of the focus population ranging from -0.478 to 0.432. As ScatR was positively correlated with SAR (Fig. S6a), a gradient of SAR values can be observed after performing simulations initialized with these initial distributions. Therefore, 89100 simulations were performed to build the link among these three parameters, in which each simulation was initialized with one of the defined initial distributions, as well as the pre-defined GroR and InifR values. After simulations, SAR values of the focus population were calculated, ranging from -5.248 to 4.648, and AbunR, as well as final competition outcomes, were also calculated and recorded. Two three-dimensional scatter plots (Fig. 5b and Fig. 5c) were then generated to visualize their relations using the *plot3D* package (v1.3) in R 4.0.2 (<https://cran.r-project.org/>).

However, higher initial abundance also potentially leads to more seeding positions for the population at the beginning, and hence resulted in an additional advantage from ‘Space Accessibility’. To eliminated the effect of initial cell number on the ‘Space Accessibility’, deriving from the difference in seeding positions at the beginning, a more general parameter perSAR was defined to quantify the asymmetry of ‘Space Accessibility’ between the focus population to its competitor, which was calculated by

$$perSAR=log(\frac{\frac{\sum_{t=0}^{t=t_{2}} {SA}_{1,t}}{{Inif}_{1}}}{\frac{\sum_{t=0}^{t=t_{2}} {SA}_{2,t}}{{Inif}_{2}}})$$

Here, ${Inif}_{1}$ represents the initial cell number of the focus population, while ${Inif}_{2}$ represents the initial cell number of its competitor. According to this definition, when the population has the same initial cell number as its competitor (that is, ${Inif}_{1}={Inif}_{2}$), perSAR equals SAR. When the difference in initial cell numbers is considered, the perSAR indicates the ratio of the ‘Space Accessibility’ normalized by the initial cell number. Analyses about perSAR were performed as the protocol described above.

**S2 Robustness test of the effect of ‘Space Accessibility’**

In our basic model, we controlled initial conditions to simplify the mathematical analysis. Then, we performed a robustness test to investigate whether the effect of ‘Space Accessibility’ on outcomes of spatial competition is statistically significant under various initial conditions.

Firstly, to test the effect of the initial number of colonized cells, we performed additional simulations using the same protocol as our basic model except for setting a gradient of initial total cell numbers of the two competing populations, ranged from 1% to 50% of the maximum population size (that is, from 4 cells to 200 cells; Table S1), while the initial ratio of the two populations was still set as 1:1. After these simulations, we performed a similar correlation analysis as before and calculated the effect size index (Cohens’D [1]) to assess whether ‘Space Accessibility’ has a significant impact on the competition outcome under different initial conditions. As shown in Table S1, we found that when the initial total number of the two populations did not exceed 10% of the maximum population size, the ‘Space Accessibility’ was significantly (P<0.001, Cohens’D>0.2 [2]) correlated with the competition outcome. In contrast, when the initial cell number was over this threshold, this effect became less significant (Cohens’D<0.2 [2]). This shift was attributed to the decreased amount of initial free positions.

Then, we tested whether the effect of ‘Space Accessibility’ on competition outcomes is different when the spatial competition occurred between two faster-growing populations, or between two slower-growing populations. We changed our basic model settings to consider three different growth rates, 0.01, 0.1, and 1 (Table S1). For each simulation, we assigned the same growth rate to two competing populations and performed correlation analyses after all the simulations. The results showed that the effect of ‘Space Accessibility’ was significant in competition between both faster-growing and slower-growing populations (Table S1).

Finally, to investigate whether the effect of ‘Space Accessibility’ is still significant when the size of space becomes larger, we simulated two populations competing for space in a larger discrete grid box of a 100×100 array. A new C++ code was written to implement these new simulations (<https://github.com/Neina-0830/BacGo-model>). Additional simulation results showed that the AbunR was still strongly positively correlated with the ‘Space Accessibility’ asymmetry, SAR (Fig. S7a; R^2^=0.885, P<0.001). Furthermore, SAR values of these simulations in which the focus population won, were still significantly higher than that for the population lost (Fig. S7b; t-value= 3.240, P< 0.01). These results indicated that larger space size also didn’t change the effect of ‘Space Accessibility’ on outcomes of spatial competition.

Together, these analyses demonstrated that the effect of ‘Space Accessibility’ on the competitive success of the focus population is robust within a wide range of initial conditions.

**S3 Figures and Tables**

**
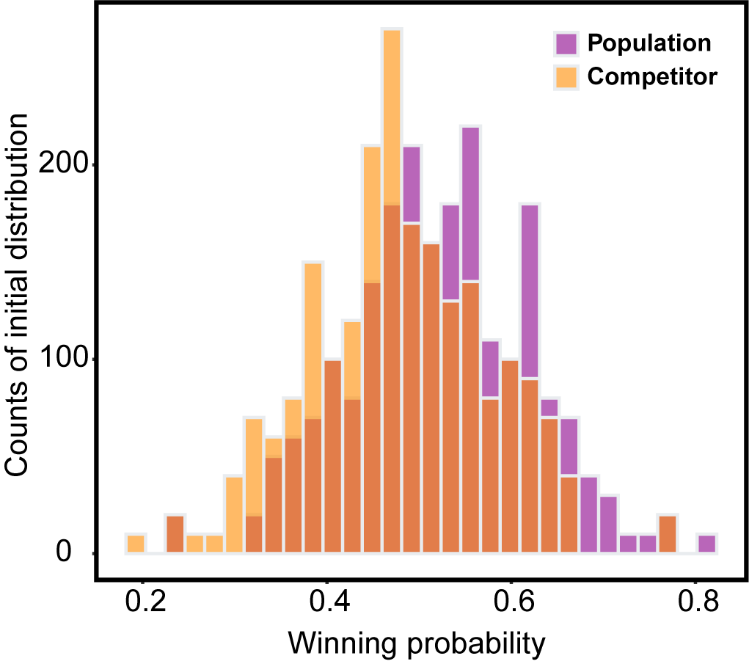
**

**Fig. S1** Histogram of the frequency distribution of winning probabilities of the focus population and its competitor. Results were summarized from 200 randomly generated initial distributions and each initial distribution was repeated 100 times to calculate the winning probability. The purple shows the histogram of the focus population. The orange shows the histograms of its competitor. The dark orange encodes the overlap between the two histograms.

**
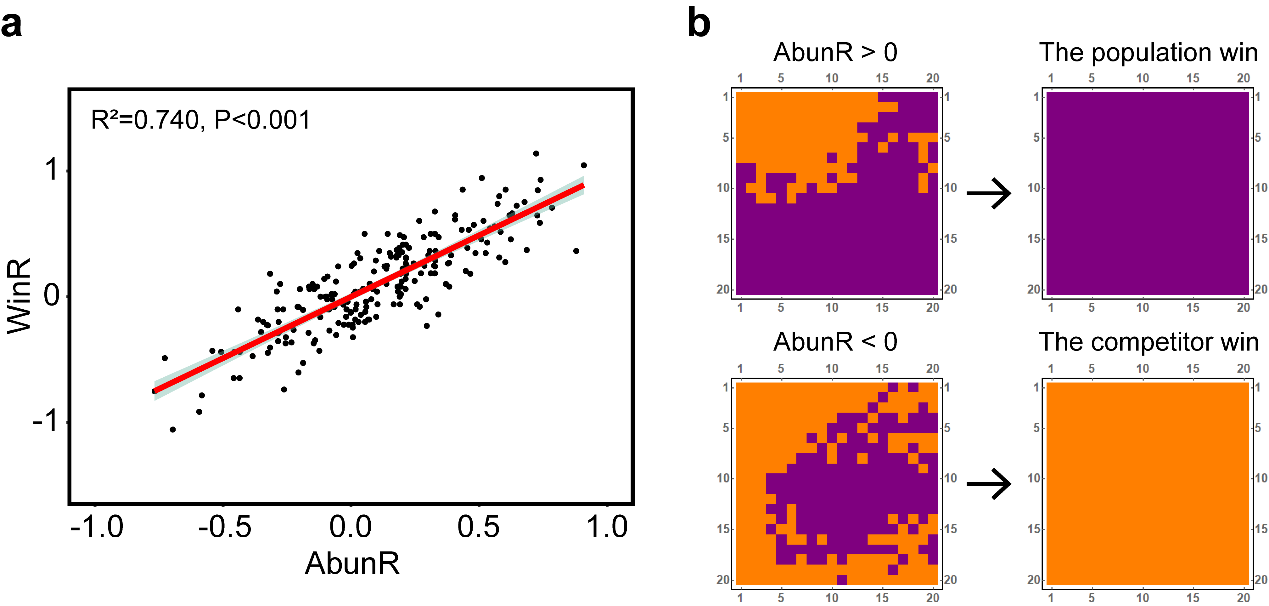
**

**Fig. S2** Correlation between AbunR and WinR. **a.** Correlation between AbunR and WinR. Results were summarized in 20000 independent simulations, which were the same as the Supplementary Figure 1. **b.** Diagram characterizing our hypothesis that the population possessing an AbunR value over zero will possess a higher probability to finally win the spatial competition.


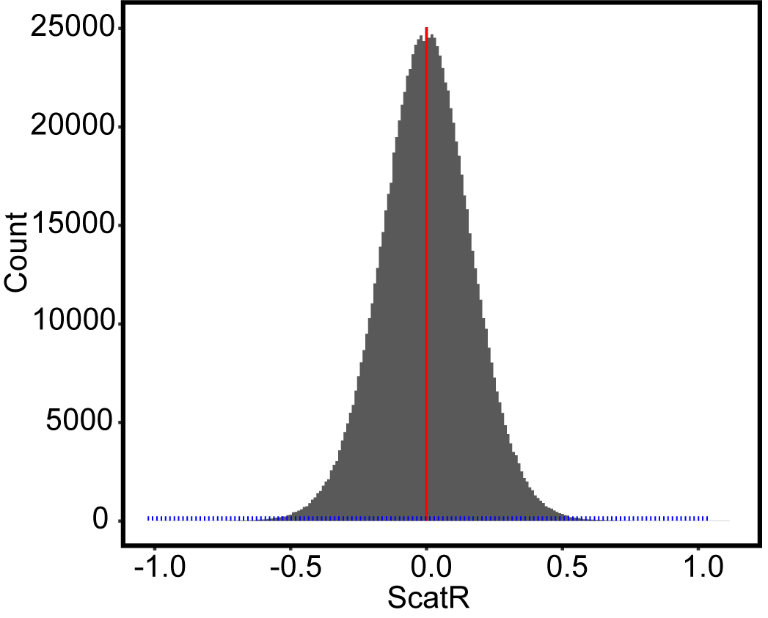


**Fig. S3** Frequency distribution histogram of ScatR with 1000000 randomly generated initial distributions. The red line is shown where ScatR equals zero, and blue lines are evenly distributed over all ranges. 363 initial distributions were selected from the red line to explore the influence of expansion freedom, and 215 initial cell distributions were selected with uniformly gradient ScatR values to explore the influence of initial scattered level, as shown in blue lines.


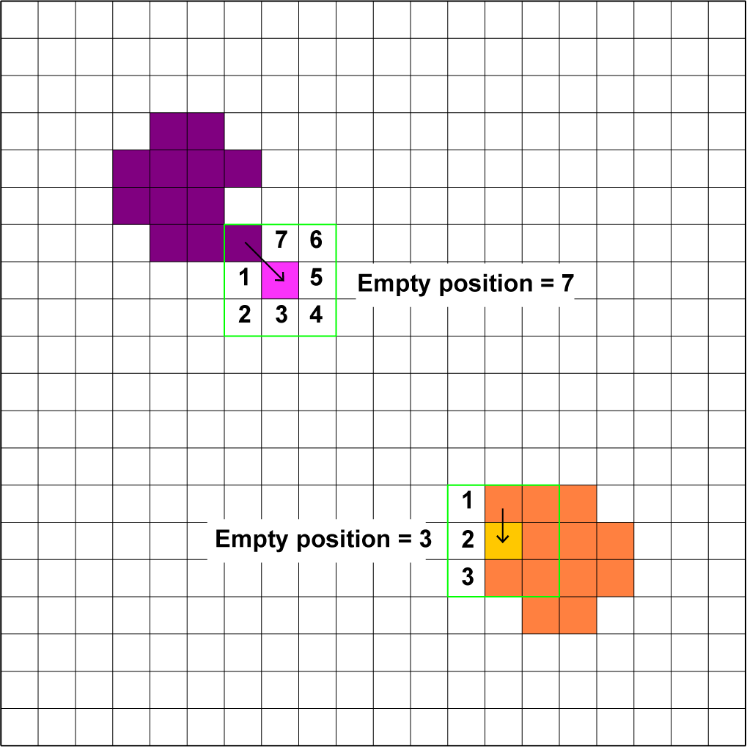


**Fig. S4** Diagram indicating the definition of ‘Empty positions’ surrounding each newly occupied position.


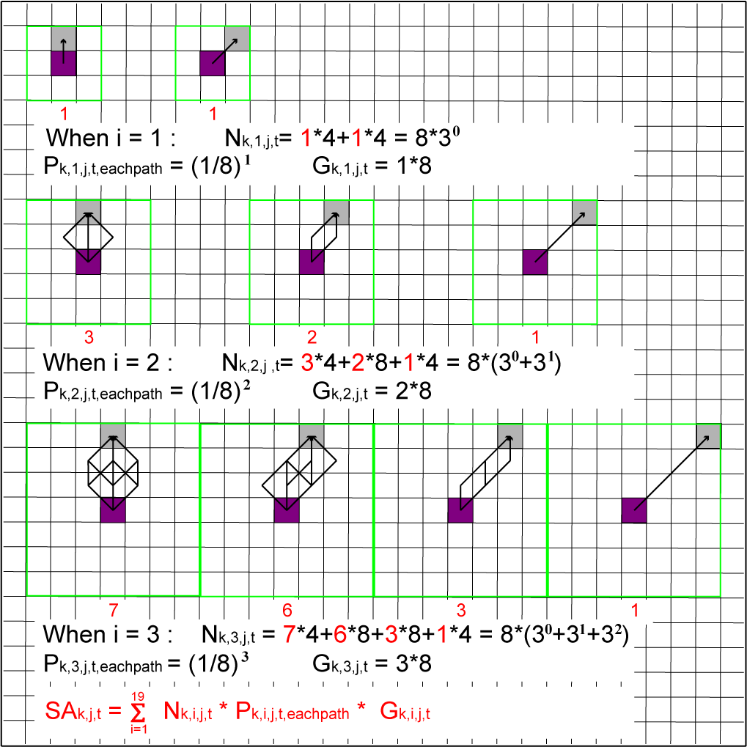


**Fig. S5** Diagrams indicating the calculation methods of ${SA}_{k, j,t}$. For clarity, we showed no occupied grids in the area, except the focus individual. As a result, $G_{k,i,j,t}$ here is the maximum number of the empty grids in the *i*th layer surrounding the *j*th individual of the *k*th population at time point *t*. When x grids are occupied in *i*th layer neighbor, $G_{k,i,j,t}$ needs to subtract x from the maximum number of empty grids.


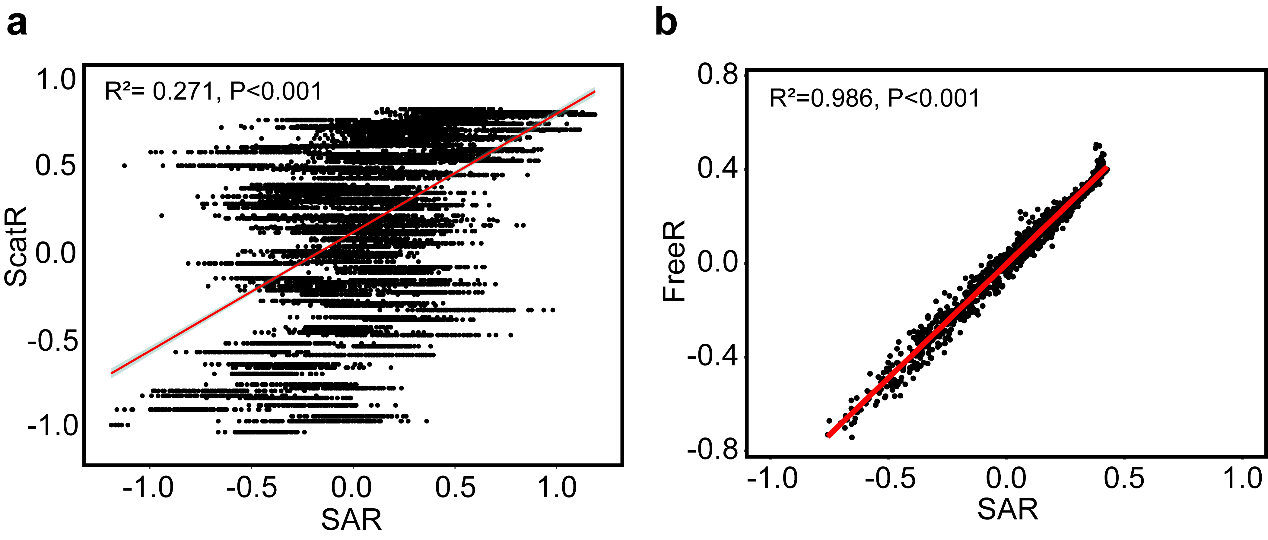


**Fig. S6** Relationship between SAR, ScatR, and FreeR. **a.** Relationship between SAR and ScatR. **b.** Relationship between SAR and FreeR. Results were summarized in 20000 independent simulations.


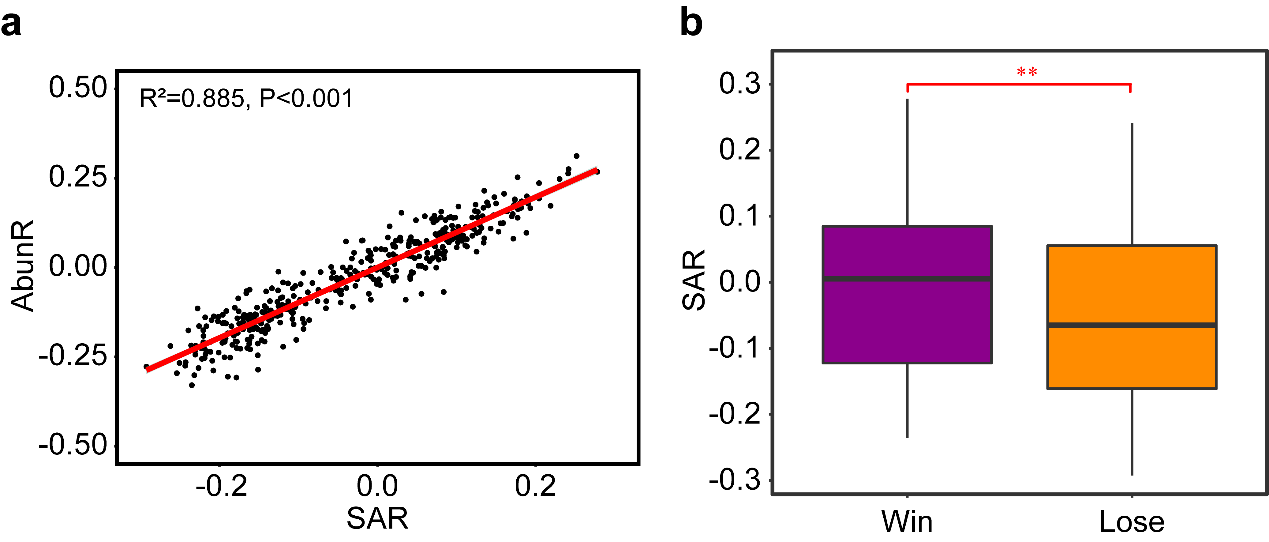


**Fig. S7** Effect of the SAR on competitive outcomes of the focus population in a space containing 100×100 array. **a.** Correlation between the ‘Space Accessibility’ asymmetry SAR of the focus population and the abundance asymmetry AbunR of the focus population. **b.** Comparison of the ‘Space Accessibility’ asymmetry SAR between simulations when the focus population won and lost. Statistical analysis was performed by a two-sample Student's t-test: **, P < 0.01. Results were summarized in 400 independent simulations.


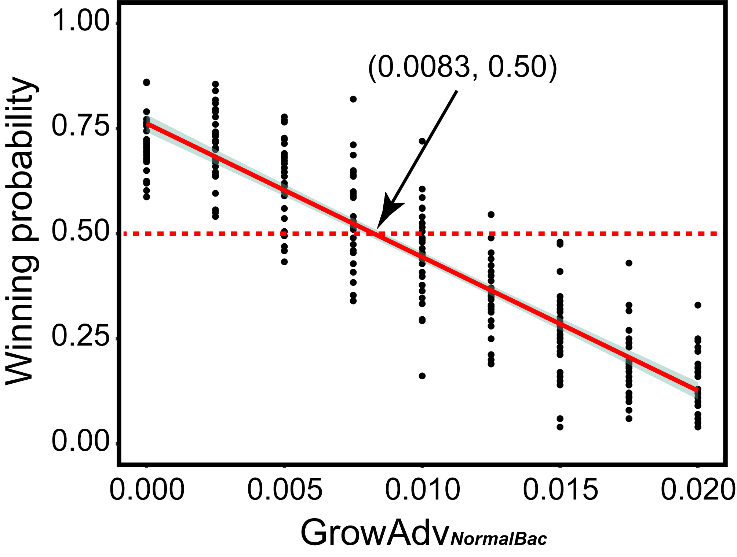


**Fig. S8** Relationship between the winning probability of SmartBac and the growth advantage of NormalBac. Results were summarized in 27000 independent simulations which were initialized by 30 random cell distributions and 9 different NormalBac growth rates. The red solid line is the winning probability curve of SmartBac and the red dotted line is the curve with a 50% winning probability. The winning probability curve of SmartBac intersects the line of 50% winning probability at (0.0083, 0.5).


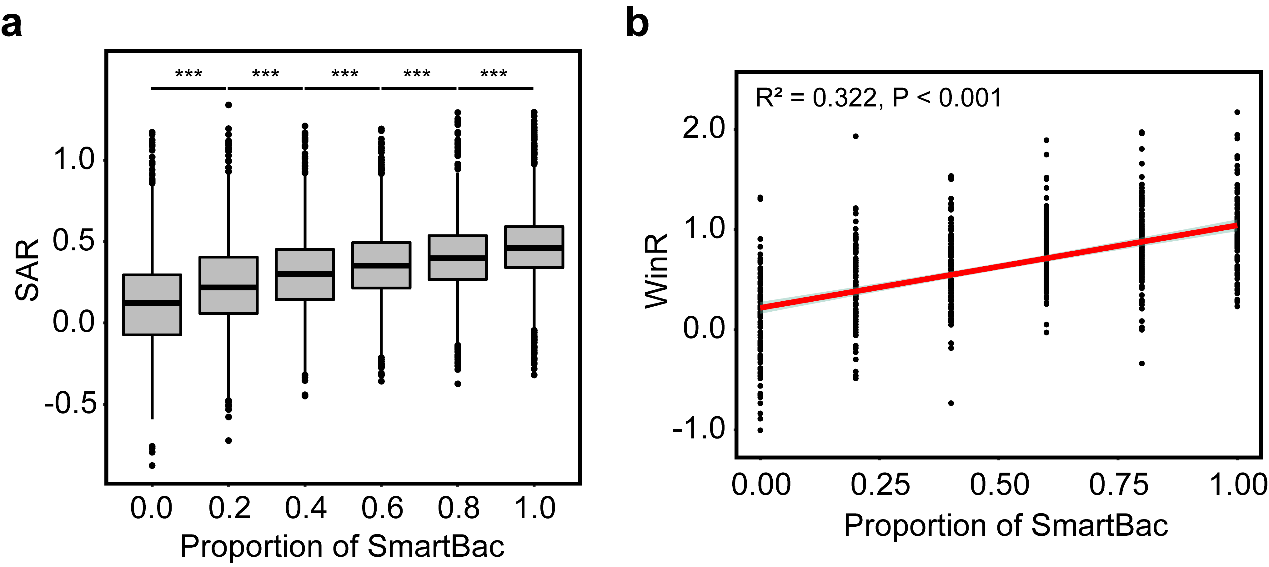


**Fig. S9** A higher proportion of SmartBac helps the focus population to win the space competition. **a.** Comparation of the ‘Space Accessibility’ asymmetry SAR between simulations when the focus population had different proportions of SmartBac. Statistical analysis was performed by a two-sample Student's t-test: ***, P < 0.001. **b.** Correlation between the proportion of SmartBac and WinR of the focus population. Results were summarized in 10000 independent simulations which were initialized by 100 random cell distributions.


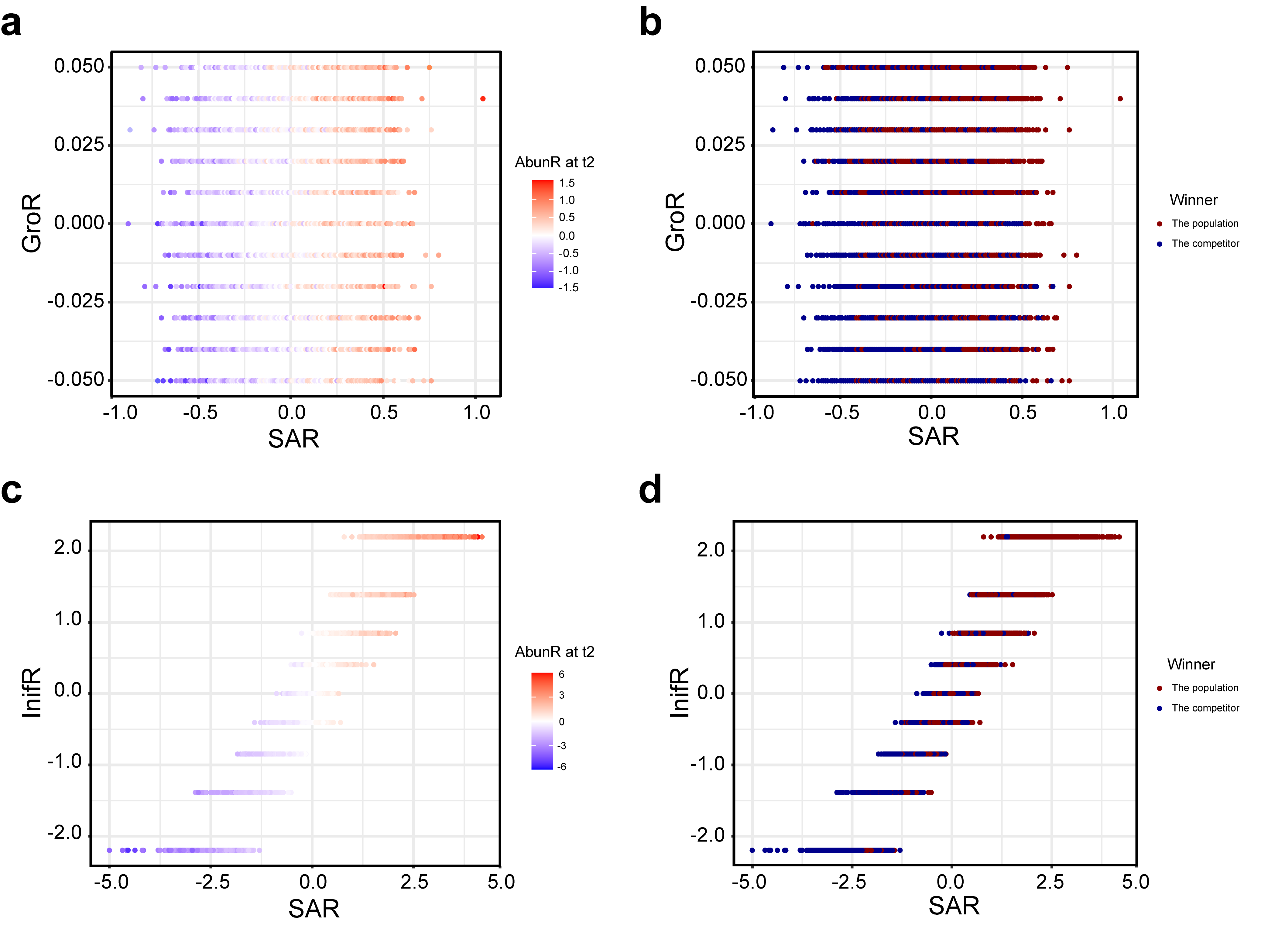


**Fig. S10** Comparison of the effect of space colonization manners with the effect of varied growth rate (a-b), in which InifR=0. Comparison of the effect of space colonization manners with the effect of initial abundance (c-d), in which GroR=0.

**
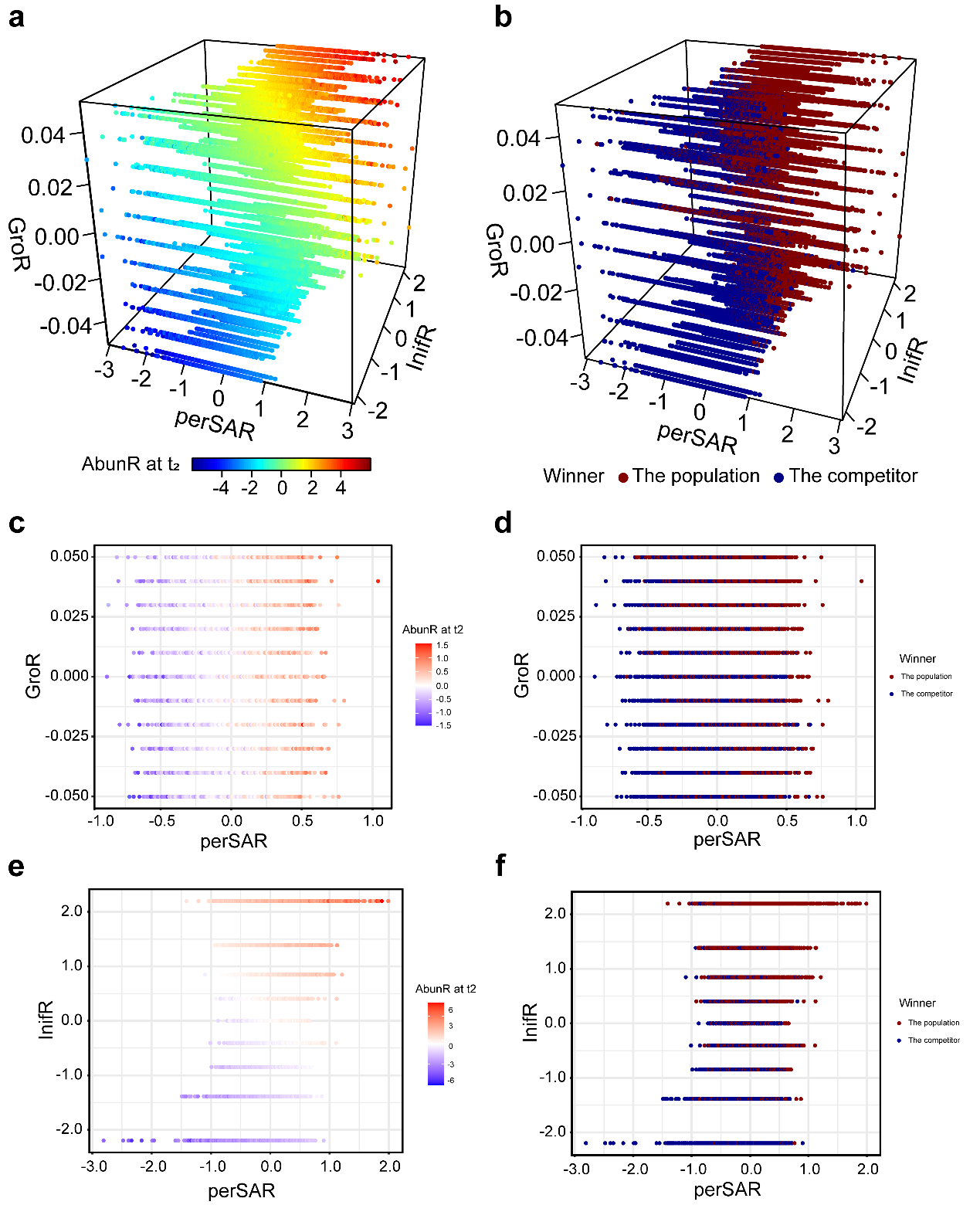
**

**Fig. S11** Comparison of the relative importance of perSAR, GroR, and InifR for outcomes of microbial competition. Values of AbunR (**a**), as well as the final competition outcomes (**b**), were recorded to estimate how these three factors collectively affect the microbial competition. Comparison of perSAR and GroR at InifR=0 (c-d). Comparison of perSAR and InifR at GroR=0 (e-f).


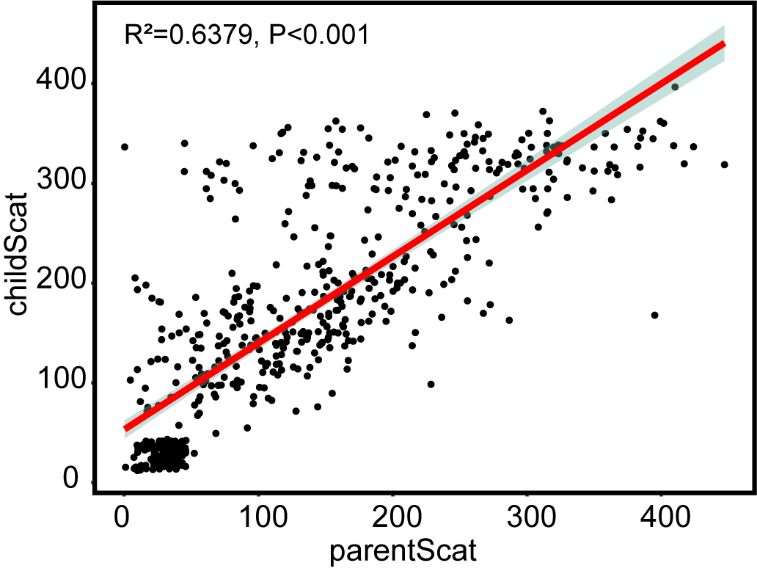


**Fig. S12** Scattered index of child trees positively correlated with that of parent trees. Data were collected from ForestGEO (<https://forestgeo.si.edu/>). ParentScat was defined as the average scattered index of these individuals whose DBH was in the top 10% for each population, and childScat was defined as the average scattered index of other 90% individuals in this population. All calculations were implemented by Wolfram Mathematica 12.0 (data and codes were available on <https://github.com/Neina-0830/BacGo-model>).

**Table S1** Summary of model variables

| **Variables** | **Definition** | **Units** | **Values** | **References** |
| --- | --- | --- | --- | --- |
| $\boldsymbol{B}_{\boldsymbol{i}}$ | Carbon biomass of the individual in the *i*th grid. | fg |  |  |
| $\boldsymbol{B}_{\boldsymbol{0}}$ | Initial C biomass of an individual. | fg | 150 | [3] |
| $\boldsymbol{\mu}_{\boldsymbol{i}}$ | Growth rate of the individual in the *i*th grid. | fg/fg∙min | 0.1 | [3] |
| $\boldsymbol{d}_{\boldsymbol{i}}$ | Random death rate of the individual in the *i*th grid. |  | 1×10^−4^ | [4] |
| $\mathbf{(}\boldsymbol{x}_{\boldsymbol{ki}}\mathbf{,}\boldsymbol{y}_{\boldsymbol{ki}}\mathbf{)}$ | Position coordinate of the *i*th individual of the *k*th population. |  | $0\leq x_{ki},y_{ki}<20$ |  |

**Table S2** Summary of the defined index

| **Defined index** | **Definition** |
| --- | --- |
| $\boldsymbol{WinR}$ | Asymmetry of winning probability between the focus population and its competitor. |
| $\boldsymbol{AbunR}$ | Asymmetry of relative abundance at t_2_ between the focus population and its competitor. |
| $\boldsymbol{ScatR}$ | Asymmetry of the scattered level of the cell distribution at t_1_ between the focus population and its competitor. |
| $\boldsymbol{FreeR}$ | Asymmetry of expansion freedom between the focus population and its competitor. |
| $\boldsymbol{SAR}$ | Asymmetry of ‘Space Accessibility’ between the focus population and its competitor. |
| $\boldsymbol{perSAR}$ | The new asymmetry index of ‘Space Accessibility’ between the focus population and its competitor. |
| $\boldsymbol{GroR}$ | Asymmetry of growth rate between the focus population and its competitor. |
| $\boldsymbol{InifR}$ | Asymmetry of relative abundance at t_1_ between the focus population and its competitor. |
| $\boldsymbol{GrowAdv}_{\boldsymbol{NormalBac}}$ | The growth rate advantage of NormalBac relative to SmartBac. |

**Table S3** Summary of symbol

| **Symbol** | **Definition** |
| --- | --- |
| $\boldsymbol{smartBac}$ | Population whose daughter cells always non-randomly select the position to ensure a higher ‘Space Accessibility’. |
| $\boldsymbol{normalBac}$ | Population whose daughter cells always randomly select the position. |
| $\mathbf{SmartGo}$ | Spatial competition model of SmartBac and NormalBac. |
| $\mathbf{SA}$ | ‘Space Accessibility’ of the population. |
| $\mathbf{Win}$ | Simulations when the focus population wins the competition. |
| $\mathbf{Lose}$ | Simulations when the focus population loses the competition. |
| $\boldsymbol{t}_{\boldsymbol{1}}$ | Time point when the competition is beginning. |
| $\boldsymbol{t}_{\boldsymbol{2}}$ | Time point when space is fully occupied. |
| $\boldsymbol{t}_{\boldsymbol{3}}$ | Time point when the winner occupies the entire space. |

**Table S4** Robustness test of the effect of ‘Space Accessibility’ on competition outcomes.

| **Initial numbers^a^** | **Growth rate** | **Simulation times** | **Determination^b^ coefficient (R^2^)^**^** | **Effect size^c^ (Cohens’D) ^***^** | **P value** |
| --- | --- | --- | --- | --- | --- |
| 100+100 | 0.1 | 1000 | 0.163 | 0.107 | <0.001 |
| 90+90 | 0.1 | 1000 | 0.137 | 0.235 | <0.001 |
| 80+80 | 0.1 | 1000 | 0.253 | 0.141 | <0.001 |
| 70+70 | 0.1 | 1000 | 0.268 | 0.141 | <0.001 |
| 60+60 | 0.1 | 1000 | 0.293 | 0.190 | <0.001 |
| 50+50 | 0.1 | 1000 | 0.314 | 0.062 | <0.001 |
| 40+40 | 0.1 | 1000 | 0.369 | 0.133 | <0.001 |
| 30+30 | 0.1 | 1000 | 0.520 | 0.092 | <0.001 |
| 20+20 | 0.1 | 1000 | 0.630 | 0.238 | <0.001 |
| 10+10 | 0.1 | 1000 | 0.614 | 0.235 | <0.001 |
| 2+2 | 0.1 | 1000 | 0.894 | 0.573 | <0.001 |
| 10+10 | 0.01 | 5000 | 0.716 | 1.022 | <0.001 |
| 10+10 | 0.1 | 5000 | 0.889 | 0.575 | <0.001 |
| 10+10 | 1 | 5000 | 0.850 | 0.431 | <0.001 |

Note:

**a.** Two same numbers in the first column denote the initial cell numbers of two competitive populations.

**b.** The determination coefficient R^2^ refers to the correlation strength of ‘Space Accessibility’ asymmetry SAR and abundance asymmetry AbunR at the "full occupied" time (t_2_).

**c.** Considering that significance level (p-value) is easily affected by sample size, we also calculated the effect size index (Cohens’D [1]) for the purpose of statistical analyses. Only if Cohens’D was greater than 0.2 [2] would SAR be considered to have an impact on AbunR.

**Table S5** Results of multiple linear regression analysis of AbunR with GroR, SAR, and InifR.

| **Parameters** | **Coefficients** | | **P-value** | **VIF** | **Adjusted R^2^** |
| --- | --- | --- | --- | --- | --- |
| GroR | | 55.393 | <0.001 | 1.007 | 0.993 |
| InifR | | 1.027 | <0.001 | 1.008 |  |
| perSAR | | 1.027 | <0.001 | 1.000 |  |

**SI References**

1. J, C. STATISTICAL POWER ANALYSIS FOR THE BEHAVIORAL-SCIENCES Percept. *Mot. Skills*, 67: 1007-1007 (1988).
2. de Paula T, et al. Acute Effect of Aerobic and Strength Exercise on Heart Rate Variability and Baroreflex Sensitivity in Men With Autonomic Dysfunction. *J Strength Cond Res,* 33(10): 2743-2752 (2019).
3. DK, B. Nutrient Uptake by Microorganisms according to Kinetic Parameters from Theory as Related to Cytoarchitecture. *Microbiol Mol Biol Rev,* 62: 636-645 (1998).
4. Allison, S. D. Cheaters, diffusion and nutrients constrain decomposition by microbial enzymes in spatially structured environments. *Ecology Letters,* 8: 626-635 (2005).
